# Development of a Simple, Underivatized Method for Rapid Determination of Free Amino Acids in Honey Using Dilute-and-Shoot Strategy and Liquid Chromatography-Tandem Mass Spectrometry

**DOI:** 10.3390/molecules27031056

**Published:** 2022-02-04

**Authors:** Wen Ma, Bingxin Yang, Jun Li, Xianjiang Li

**Affiliations:** 1 State Key Laboratory of Natural and Biomimetic Drugs, School of Pharmaceutical Sciences, Peking University, Beijing 100191, China; wen.ma@bjmu.edu.cn (W.M.); lijun@bjmu.edu.cn (J.L.); 2 Key Laboratory of Chemical Metrology and Applications on Nutrition and Health for State Market Regulation, Division of Metrology in Chemistry, National Institute of Metrology, Beijing 100029, China; yang.bingxin@outlook.com

**Keywords:** free amino acid, hydrophilic interaction liquid chromatography, dilute-and-shoot, honey sample

## Abstract

A simple, fast and reliable analytical method was developed for 20 free amino acids (FAAs) determination in honey samples through a dilute-and-shoot strategy and hydrophilic interaction liquid chromatography tandem mass spectrometry. Compared with previous reports, direct dilution by water has significantly reduced the matrix effect and facilitated full extraction of FAAs. Further, a 5 min determination method was established with an acetonitrile–water mobile phase system with 0.1% formic acid addition. The established method was validated and demonstrated several advantages including short detection time, wide linear range over 3–4 orders of magnitude, high sensitivity down to 0.1 ng/mL and negligible matrix effect. Twenty FAAs were determined in 10 honey samples from different botanical origins by this method, and 19 FAAs were found. This general applicable method was also promising for fast determination of FAAs in other practical samples.

## 1. Introduction

As an extremely valuable food product, honey has also been considered to have medicinal effects including anti-inflammatory, anti-tumor and anti-oxidative activity [1,2]. Honeys from different origins could be used to treat different diseases. For example, buckwheat honey has been reported to treat cardiovascular and nervous diseases [3], whereas linden honey can serve as a diuretic, choleretic and disinfectant [4]. Traditional quality control methods for honeys are based on their physicochemical characteristics such as moisture content, sum of carbohydrates (glucose and fructose), diastase activity and even electrical conductivity property [5,6]. Recently, the quantitative profile of free amino acids (FAAs) in honey has aroused increasing attention because it provides information to identify the botanical and geographical origin of honeys and is helpful for authenticity verification [7,8].

The development of analysis methods for FAAs has been a long historical project and continuous innovation and improvement have been achieved in this field. The accurate detection of FAAs not only facilitates nutritive composition analysis in food, but also provides valuable information for elucidating disease machinery. Several studies have reported FAAs analysis using ion-exchange chromatography [9], capillary electrophoresis [10], gas chromatography [11] and high-performance liquid chromatography [12]. Owing to their high polarity and absence of specific chromophores, a derivatization step is usually required for FAAs before these analysis methods to improve detection sensitivity [13,14]. For example, a fully automated FAAs analyzer, which is frequently applied today, contains ion-exchange chromatography separation, post-column derivatization using ninhydrin reagent and ultraviolet detection under 570 nm or 440 nm. However, derivatization steps usually suffer from long sample preparation time, incomplete and instable derivatization and by-product interference. Moreover, the use of costly and potentially toxic reagents, tedious derivatization procedures and decreased reproducibility are also unavoidable drawbacks of derivatization.

Liquid chromatography tandem mass spectrometry (LC-MS/MS) is undoubtedly a powerful tool for trace analyte detection due to its strong qualitative and quantitation ability [15,16]. By monitoring characteristic ion pairs of each analyte, LC-MS/MS allows one to detect targeted molecules with high specificity and sensitivity even in complex matrices [17,18,19]. Therefore, the use of the LC-MS/MS technique could eliminate the inherent drawbacks of FAA derivatization. The first successful works in LC-MS and LC-MS/MS on FAA analysis were reported in 1999, using ion-pairing reversed phase liquid chromatography (IP-RPLC) [20,21,22]. Since then, this approach has been widely applied in the quantification of FAAs in many biological and food matrices. To date, several approaches have been made for underivatized FAAs analysis coupled with either hydrophilic interaction liquid chromatography (HILIC) [23] or RPLC [24]. While LC-MS/MS has been used in FAAs determination, it still faces big challenges such as long chromatographic separation time, limited linear range, unsatisfactory sensitivity and poor isomer separation performance [25,26]. Additionally, full analysis of 20 natural FAAs with a simple, fast and underivatized method has rarely been reported. On the other hand, for honey sample preparation, solid-phase extraction (SPE) is one of the most used methods, but it suffers from complex procedures, high cost and relatively low recoveries. In comparison, the dilute-and-shoot strategy provides a simple and easy-to-operate method for solid samples [27] that uses a suitable solvent to extract analytes into liquid phase, followed by dilution before determination. Adequate dilution reduces the matrix effect and simplifies the further clean-up procedure. However, the dilute-and-shoot strategy coupled with LC-MS/MS for rapid determination of 20 natural FAAs in honey samples has not been reported.

In this work, we introduced a dilute-and-shoot strategy for honey sample preparation and developed a simple, underivatized, and reliable analytical method for rapid determination of 20 natural FAAs in honey samples. Diluent solvent in sample preparation and LC conditions including type of chromatographic columns, mobile phases and additives were carefully optimized to provide an accurate and fast analytical method. The applicability of the method was tested on several honey samples from different botanical origins. This developed LC-MS/MS method was also general applicable for rapid determination of FAAs from other real samples.

## 2. Results and Discussion

### 2.1. Dilute-and-Shoot Strategy Optimization

#### 2.1.1. Type of Dilution Solvent

**Figure 1:**
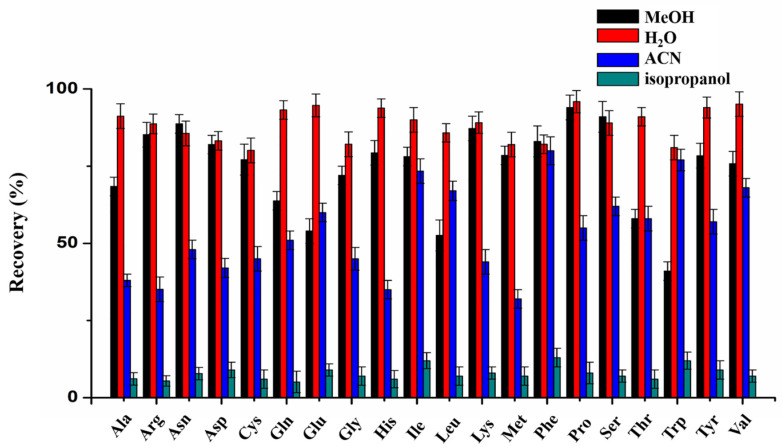
Effect of different extraction solvents on the recovery.

Dilution solvent was the first parameter to optimize because it has a significant impact on the final result. Four solvents, water, methanol (MeOH), acetonitrile (ACN) and isopropanol, were selected to dilute the matrix and extract FAAs. As shown in Figure 1, among these four solvents, acetonitrile and isopropanol had poor extraction recovery, in that some FAAs could not be extracted and showed low MS signal, and water performed the best according to the dramatic increase in recovery. This is because FAAs have high polarity, so a polar solvent can fully extract FAAs and generate higher signal intensity. For two polar solvents, water and MeOH, we also carefully measured the extraction efficiencies of the mixed solvent with different proportions (volume ratio of MeOH from 30% to 70%) compared with pure water or pure MeOH. The results in Figure S1 show that there was a decrease in extraction efficiency with the increasing amount of MeOH. Therefore, water was an ideal extraction solvent compared with others.

#### 2.1.2. Acid Addition Effect

Some studies have reported that the addition of acid could facilitate extraction and generate higher signals. Here, different acid additives (Formic acid (FA), HCl, CH_3_COOH with volume ratio 0.2%) were added into water, respectively, to test the acid addition effect. The results in Figure S2 show that pure water generally led to a higher recovery than the acidic water. However, when using HCl added to water as an extraction solvent, the retention time of all the FAAs was totally changed, and the shape of the chromatographic peak of some FAAs (such as phenylalanine (Phe)) became wider and tended to split. Therefore, pure water was finally selected as the extraction solvent.

#### 2.1.3. Effect of Dilution Fold

**Figure 2:**
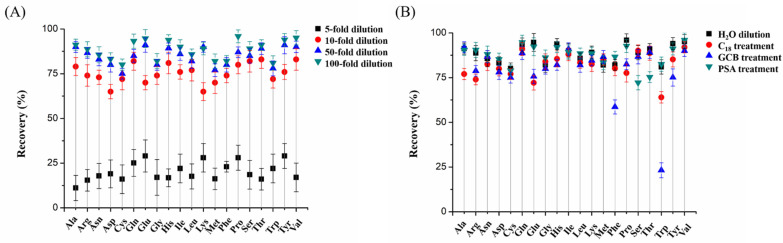
Effect of (**A**) dilution fold and (**B**) dSPE treatment on the recovery.

Honey contains a high content of carbohydrates (60%–80% in mass ratio) and small amounts of FAAs (less than 1%). During the water extraction process, some carbohydrates are extracted simultaneously. To reduce the matrix effect and loss of analytes, direct dilution was used. For the 100 mg honey sample, different amounts of water (0.5 mL, 1 mL, 5 mL, 10 mL) were added to extract FAAs. The dilution factor was 5-fold, 10-fold, 50-fold and 100-fold, respectively. The signal intensity of internal standard (IS) between calibration solvent and diluted honey sample was determined, and recovery of IS was used as an indicator to measure the matrix effect. Figure 2A showed that the recovery dramatically increased with the amount of water added, and relative standard deviations (RSDs) were decreased. For sample preparation, 0.5 mL was enough for full extraction, and a significant matrix effect was observed. In the dilute-and-shoot strategy, the employed dilution factors usually ranged from 2 to 50, according to the matrix. Here, 100-fold water was used to extract FAAs and dilute the honey sample; as a result, the recovery of IS was higher than 90%, and reproducibility was also improved, which demonstrated the matrix effect was negligible at this condition, and 10 mL was enough to fully extract FAAs.

#### 2.1.4. Dispersive SPE

The dispersive SPE (dSPE) procedure is one of the most-used sample preparation methods for honey samples. The use of dSPE sorbents could efficiently remove interference from other substances such as carbohydrates, pigments or proteins. Here, three commonly used sorbents including C_18_, graphitized carbon black (GCB) and primary secondary amine (PSA) were tested. After being diluted by water, 10 mg sorbent was added into the extraction solvent, and the mixture was incubated for 30 min. After centrifugation, the supernatant was injected into the LC-MS/MS system, and the results were compared with pure water extraction. As shown in Figure 2B, after carefully comparing each FAA with or without dSPE sorbents treatment, we concluded that the extraction efficiency was comparable between pure water and PSA clean-up. This may be ascribed to the reason that 100-fold dilution by water was an efficient way to reduce the matrix effect and extract FAAs, which was comparable to the PSA clean-up procedure (PSA tended to remove some polar substances such as carbohydrates while also adsorbing some hydrophilic FAAs). However, C_18_ and GCB showed some negative effect on the extraction because they had interactions with some hydrophobic FAAs (such as Phe and tryptophan (Trp)). According to the result, and considering this time-consuming operation, the potential loss of FAAs caused by sorbent adsorption, no sorbent was used in the final extraction process.

### 2.2. Liquid Chromatography Tandem Mass Spectrometry

For FAA analysis, both RPLC and HILIC methods have been explored in previous reports. However, it still faces many challenges. First, because FAAs are polar and hydrophilic analytes, RPLC usually showed low or no retention for hydrophilic FAAs, causing the majority of FAAs eluted within void time or co-eluted with polar interferences. Second, some isomers such as isoleucine (Ile) and leucine (Leu) and FAAs that have almost the same MRM transition, such as glutamine (Gln) and lysine (Lys), could not be totally separated in some LC conditions, resulting in poor peak shapes and inaccurate quantitation results. Third, relatively long chromatographic separation time was required (usually over 20 min) for full analysis of 20 FAAs, which limited high-throughput and fast determination applications. To develop a simple, fast and underivatized method to determine 20 FAAs, several parameters including characteristic ion pairs in MS, type of chromatographic column, mobile phase, acid additive and final gradient were fully investigated.

#### 2.2.1. Characteristic MRM Transition of Each FAA

In LC-MS/MS, MRM scan mode is a gold standard for accurate quantitation. It selects the characteristic precursor ion of each FAA in the first quadrupole, followed by the collision in the second quadrupole to produce the product ions, and the characteristic product ions are selected in the third quadrupole. Due to the two-round selection, MRM transition of each FAA is highly specific and offers high detection specificity and sensitivity in complex matrices. Therefore, MRM transitions of 20 FAAs were measured for accurate quantitative analysis (typical transition spectra were provided in the Figure S3). Particularly, isomers Leu and Ile were distinguished using different transitions, and Gln and Lys were separated by LC. MS-related results including MRM transitions, declustering potential (DP) and collision energy (CE) for each FAA are listed in Table S1.

#### 2.2.2. Chromatographic Column in LC separation

**Figure 3:**
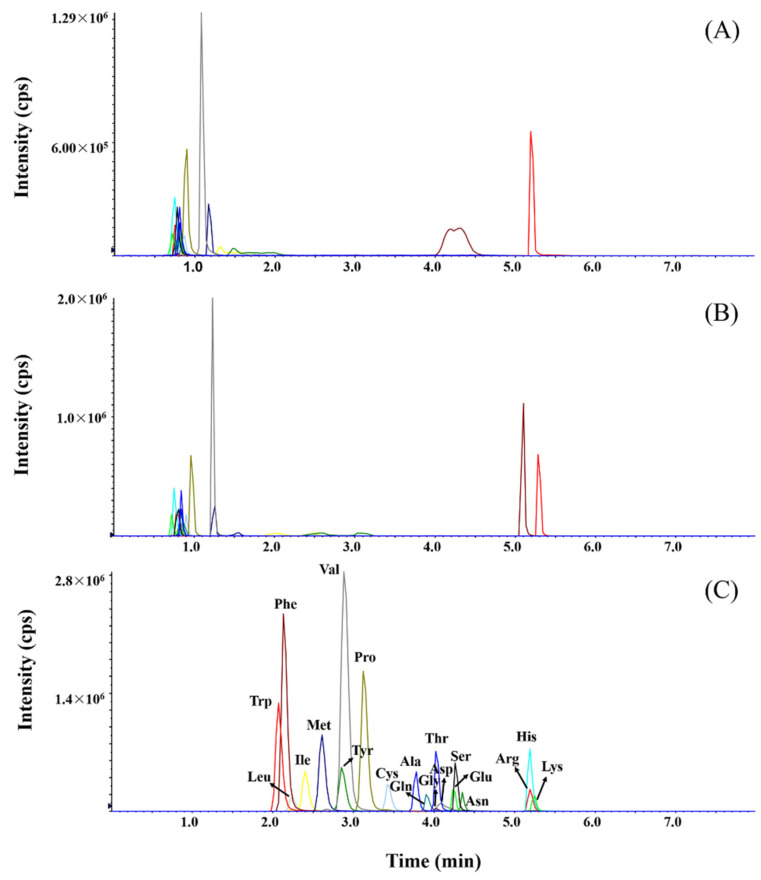
Chromatographic performance of three columns, (**A**) Polar C_18_, (**B**) HSS T3 and (**C**) BEH amide for 20 FAAs separation at the same concentration. Each peak was annotated in (**C**), and the same color represents the same amino acid.

The selection of chromatographic column is a predominate factor that directly influences the separation performance. Considering FAAs are polar and hydrophilic, three columns including Polar C_18_, HSS T3 and BEH amide column were tested. Compared with classic C_18_ column, Polar C_18_ and HSS T3 were considered to have higher retention towards polar analytes. The gradients for each column are listed in Table S2. As shown in Figure 3, which gives a direct comparison of these three columns, most FAAs still showed poor retention on Polar C_18_ and HSS T3 columns. Although the initial mobile phase was set at 98% H_2_O and was retained for 2 min, more than 10 FAAs were eluted within 1 min. This caused the poor separation and peak shape for isomers (Figure S4) and co-elution of other polar interferences. In contrast, the BEH amide column showed proper retention for all 20 FAAs, and isomers were fully separated in the whole gradient. Therefore, an HILIC method based on a BEH amide column was selected for the analysis of 20 FAAs.

#### 2.2.3. Mobile Phase and Additive in HILIC Separation

**Figure 4:**
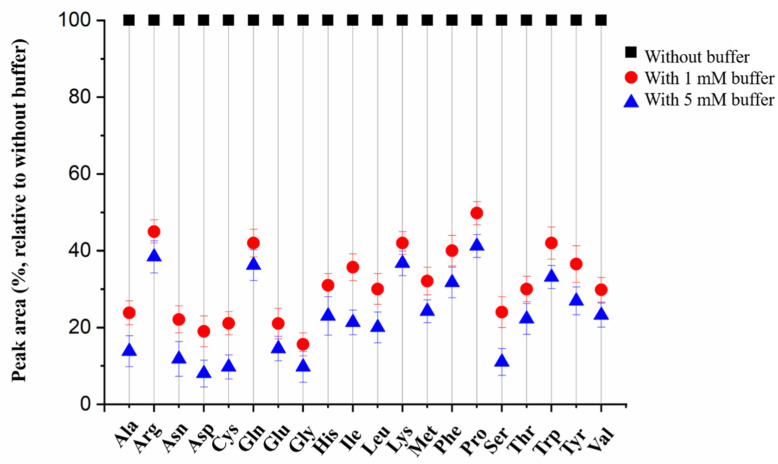
Effect of ammonium formate buffer addition in the mobile phase on the chromatographic separation performance.

Except chromatographic column, the mobile phase and additive also play vital roles for the detection specificity and sensitivity. We first tried a water/MeOH system for separation and found both water and MeOH showed strong elution ability, in that 17 FAAs were eluted within the first 2 min. Then, a water/ACN system was tested, and proper retention was achieved. In addition, the additive greatly influenced the final separation performance. For example, if no additive was added in the mobile phase, three FAAs (arginine (Arg), histine (His), Lys could not been separated within 10 min, and wider peaks were observed. When 0.1% FA was added to water and ACN, these three FAAs were acidified and could be separated within 8 min. Furthermore, we also tested the influence of ammonium formate additive, which is frequently used in LC separation to improve peak symmetry and detection sensitivity. However, the addition of ammonium formate (1 mM and 5 mM in water) resulted in a significant suppression for the MS signal from 2 times to 12 times (Figure 4), and serious tailing peaks (His and aspartic acid (Asp)) were observed. Under this condition, the MS signal was easily blended into the background, and sensitivity would dramatically decrease. This phenomenon was consistent with several previous reports [28,29]. Therefore, only 0.1% FA was selected as the additive. After confirming the final mobile phases, the elution gradient was carefully optimized. As a result, a simple and fast 5 min gradient was used for the determination of 20 FAAs (Table S3). The final chromatogram is shown in Figure S5. Compared with previous reports, our method provides a simple, ultrafast and reliable analytical method.

### 2.3. Method Validation

The developed method was validated by measuring several parameters including linearity, linear dynamic range (LDR), limit of detection (LOD), carry-over effect and intraday and interday RSD. Mixed IS solutions with a series of concentrations were used to determine linearity by plotting the peak area versus the concentration. The results in Table 1 showed that this method had a wide linear range over 3–4 orders of magnitude, with linearities (R^2^) ranging from 0.9963 to 0.9993. Additionally, using the criterion of a signal–noise ratio larger than 3, LODs of 20 FAAs were among 0.1–3.0 ng/mL, which showed high sensitivity compared with several other reports [30,31]. Method precision was also measured by conducting the experiment in six replications in one day or on three consecutive days. The results showed that intraday RSDs and interday RSDs were in ranges of 4.14–7.62% and 6.72–10.56%, respectively, demonstrating good precision of the established method. In order to investigate the carry-over effect, a reagent blank was injected immediately after the highest standard, and no obvious carry-over was observed. To explore the matrix effect after dilution procedure, the signal of IS between neat dilution solvent and diluted honey sample was compared, and it showed almost no difference, indicating that the matrix effect was negligible after 100-fold dilution.

### 2.4. Real Sample Analysis

The developed simple and underivatized method was further used for fast determination of 20 FAAs in 10 honey samples from different botanical origins including linden honey (2 samples), locust honey (2 samples), loquat honey (4 samples) and jujube flower honey (2 samples). Detailed sample information is listed in Table S4. After the dilute-and-shoot strategy, the extraction solvent was further diluted to initial mobile phase and injected into the HILIC-MS/MS system. For each sample, the experiment was repeated three times. Results in Table S5 showed that, except cysteine (Cys), 19 FAAs were all found in honey samples with different amounts, and Phe and proline (Pro) were the most dominated FAAs in these honeys. As one of eight essential amino acids, the content of Phe in honey was from 3.1 μg/g to 369.7 μg/g. The *p*-values of Phe content in honey from different botanical origins were summarized in Table S6, and the results show that Phe content in linden honey was significantly higher than that in other groups. It is worth mentioning that the aim of practical sample analysis was to show the application of the developed method, and further validation in larger cohorts is warranted to get a reliable statistical result. All the above result could provide information about nutritive composition analysis and give instructive advice on dietary choices.

## 3. Materials and Methods

### 3.1. Chemicals and Materials

Twenty amino acid reference standards including glycine (Gly), alanine (Ala), Phe, threonine (Thr), Lys, tyrosine (Tyr), Pro, Arg, Ile, His, Gln, glutamic acid (Glu), Cys, Trp, asparagine (Asn), Leu, methionine (Met), Asp, valine (Val), serine (Ser) and IS stable isotope labeled amino acid mixture (Product No: 909653) were all purchased from Sigma-Aldrich (St Louis, MO, USA).

Chromatography columns were Luna Omega Polar C_18_ (2.1 mm × 100 mm, 3 μm) from Phenomenex (Torrance, CA, USA), Acquity HSS T3 column (2.1 mm × 100 mm, 1.8 μm) and Acquity BEH amide column (2.1 mm × 100 mm, 2.5 μm) from Waters (Milford, MA, USA). Solid-phase extraction (SPE) sorbents including C_18_, GCB and PSA were from Agilent Technologies (Beijing, China). Ultrapure water from Milli-Q was used. FA, MeOH, ACN and isopropanol of LC-MS grade were purchased from Fisher (Waltham, Massachusetts, USA). Acetic acid (CH_3_COOH) and hydrochloric acid (HCl) were obtained from Sinopharm Chemical Reagent (Beijing, China). Ten honey samples from different botanical origins were purchased from the local market.

### 3.2. Preparation of Calibration Solutions

Stock solution of IS mixture was prepared by dissolving an accurately weighted portion of the pure standard compound in water/ MeOH (*v*/*v* = 9:1) at a concentration of 1 mg/mL. Matrix-matched calibration curve was prepared with concentrations of mixed standard between 0.5 and 5000 ng/mL (in initial LC mobile phase). All solutions were stored at −20 °C for further use.

### 3.3. Instruments

Accurate quantitation was measured in an UltiMate 3000 Ultra-HPLC system (Thermo, San Jose, CA, USA) coupled with an API 4000 QTRAP mass spectrometer (AB SCIEX, Foster City, CA, USA). Quantitative analysis was realized with multiple reaction monitoring (MRM) scan mode, and all FAAs were detected in positive ion mode in the form of proton adduct. Detailed MS parameters including precursor ion, product ion, DP and CE for 20 FAAs and IS were carefully optimized. For LC condition, three columns including reversed phase column and hydrophilic interaction column were tested, and different mobile phases and additives were assessed to obtain the best separation performance.

### 3.4. Sample Preparation

A 100 mg sample of honey was accurately weighed and mixed with 10 mL extraction solvent (100-fold dilution). A certain amount of IS was added into the mixture to obtain a final concentration of 100 ng/mL. The mixture was vortexed for 1 min to form a homogeneous solution, followed by sonication for 5 min to fully extract FAAs into the solvent. Then, the sample was centrifuged at 12,000 rpm for 10 min, and the supernatant was further diluted to 50% ACN (2-fold dilution) and injected into an LC-MS/MS system. The recovery of IS was used as an indicator for the optimization process.

On the other hand, to compare the results of the direct dilution method with SPE sorbent clean-up method, 10 mg sorbents (C_18_, GCB or PSA) were added to 10 mL extraction solvent, respectively, and the mixture was incubated for 30 min, followed by centrifugation at 12,000 rpm for 10 min. The supernatant was further diluted to 50% ACN and injected into an LC-MS/MS system.

### 3.5. Method Validation

To validate the applicability of the developed method for 20 FAAs determination, the linear range and linearity, LOD, carry-over effect, matrix effect and precision were investigated. Because there is no blank honey sample that is free of FAAs, IS containing 20 stable isotope labeled amino acids were added into honey, and a matrix-matched calibration curve was prepared. The same amount of IS was also added into the neat dilution solvent, so that the signal of IS between neat dilution solvent and diluted honey sample could be used as an indicator to evaluate the matrix effect.

### 3.6. Real Sample Analysis

Ten commercial honey samples were collected from 4 botanical origins, including linden honey (2 samples), locust honey (2 samples), loquat honey (4 samples) and jujube flower honey (2 samples). Dilute-and-shoot sample preparation was used for all samples, followed by LC-MS/MS determination. Three replicates were tested for each sample to obtain reliable results. Retention time and MRM channel were used to identify each FAA, and accurate quantification was achieved using a matrix-matched calibration curve.

## 4. Conclusions

In this study, we have developed and validated a simple and fast method for 20 FAAs determination in honey by dilute-and-shoot strategy for sample preparation and HILIC-MS/MS. The simple and direct sample preparation procedure effectively reduced the matrix effect. In addition, a fast and reliable 5 min analytical method was established using the HILIC method for fast determination of 20 FAAs in honey samples. This approach offered many advantages including simple and time-saving sample preparation, short detection time, wide detection linear range over 3–4 orders of magnitude, high sensitivity and negligible matrix effect. This generally applicable LC-MS/MS method is also promising for the fast determination of FAAs in other practical samples.

## Figures and Tables

**Table 1:** Analytical performances of the established method.

Analyte	LDR (ng/mL)	Calibration Equation	R^2^	LOD (ng/mL)	Intraday RSDs (%, *n* = 6)	Interday RSDs (%, *n* = 6)
Ala	1–3000	Y = 752 X + 755	0.9963	0.2	4.14	8.28
Arg	1–3000	Y = 483 X + 677	0.9986	0.2	5.46	9.12
Asn	2–4000	Y = 266 X + 422	0.9986	0.5	6.54	7.87
Asp	4–5000	Y = 471 X + 148	0.9974	1.0	5.12	9.22
Cys	4–5000	Y = 305 X + 91	0.9987	1.0	4.88	8.88
Gln	2–3500	Y = 602 X + 268	0.9978	0.5	4.77	7.56
Glu	2–3500	Y = 935 X + 496	0.9982	0.5	6.67	9.24
Gly	10–4500	Y = 24 X + 465	0.9979	3.0	6.82	8.71
His	1–2500	Y = 1330 X + 925	0.9984	0.2	7.62	8.28
Ile	1–3000	Y = 1050 X + 151	0.9962	0.2	5.66	7.16
Leu	2–4000	Y = 384 X + 597	0.9991	0.5	7.34	8.87
Lys	2–3000	Y = 325 X + 615	0.9989	0.5	4.65	6.72
Met	1–2500	Y = 2080 X + 102	0.9978	0.2	6.88	8.28
Phe	0.5–1500	Y = 5100 X + 976	0.9985	0.1	7.12	9.46
Pro	0.5–1500	Y = 4730 X + 741	0.9992	0.1	5.38	8.72
Ser	1–2500	Y = 831 X + 725	0.9982	0.2	4.87	7.28
Thr	1–2500	Y = 1090 X + 252	0.9989	0.2	6.78	9.12
Trp	1–2000	Y = 3060 X + 204	0.9978	0.2	7.56	10.56
Tyr	1–2500	Y = 1220 X + 349	0.9988	0.2	5.12	7.87
Val	0.5–1000	Y = 7350 X + 1410	0.9993	0.1	6.55	8.46

## Data Availability

Not applicable.

## Supplementary Materials

The following are available online, Figure S1: Influence of different extraction solvents on the recovery. Figure S2: Influence of different acid additives on the recovery. Figure S3: Representative product ion spectra of FAAs, precursor ions were highlighted in black and product ions used for quantitation were marked in red. Figure S4: Retention performance of two pairs of isomers on Polar C_18_ (upper two graphs) and HSS T3 column (bottom two graphs). Figure S5. Chromatogram of 20 FAAs using 5 min gradient elution. Table S1: MS/MS parameters for 20 FAAs. Table S2: Initial LC gradient for three columns. Table S3: Final LC condition after optimization using BEH amide column. Table S4: Detailed information of these ten honey samples. Table S5: FAA content in ten honey samples. Table S6: *p*-value of Phe content in honey from different botanical origins.

## Sample Availability

Samples of the compounds are available from the authors.
