# Supplementary material for: Development of a Simple, Underivatized Method for Rapid Determination of Free Amino Acids in Honey Using Dilute-and-Shoot Strategy and Liquid Chromatography-Tandem Mass Spectrometry
Source: Molecules. 2022 Feb 4;27(3):1056. doi: 10.3390/molecules27031056 (PMC8838828; doi:10.3390/molecules27031056)
Supplement: Supplementary file 1 [file molecules-27-01056-s001.zip › molecules-1562485-supplementary.pdf]

Supporting Information

Development of a simple, underivatized method for rapid determination of free amino acids in honey using dilute-and-shoot strategy and liquid chromatography–tandem mass spectrometry

Wen Ma,[1] Bingxin Yang,[2] Jun Li,[1] Xianjiang Li[2,*]

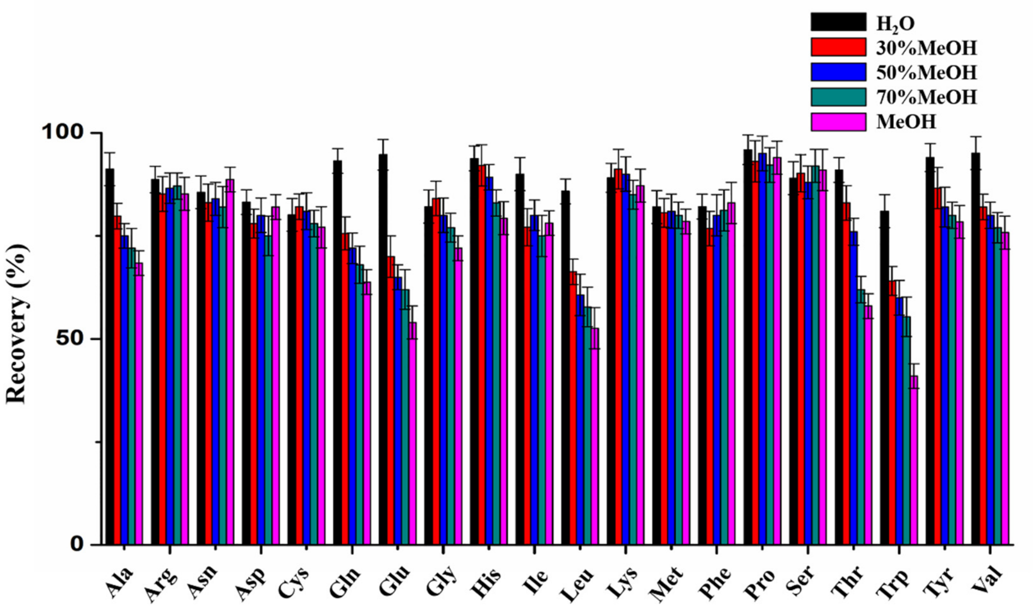

Figure S1. Influence of different extraction solvents on the recovery.

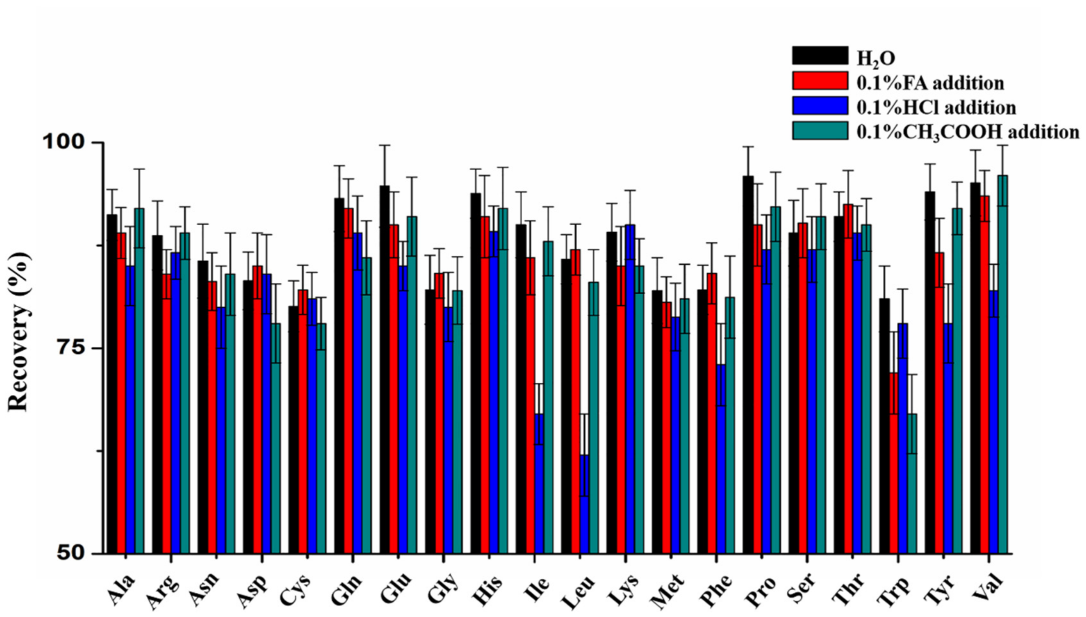

Figure S2. Influence of different acid additives on the recovery.

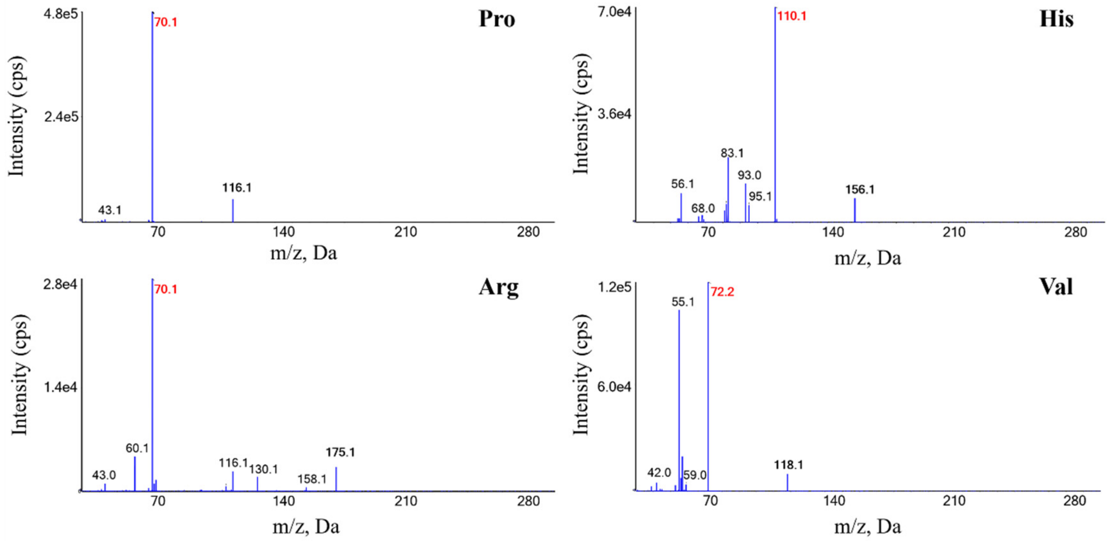

Figure S3. Representative product ion spectra of FAAs, precursor ions were highlighted in black and product ions used for quantitation were marked in red.

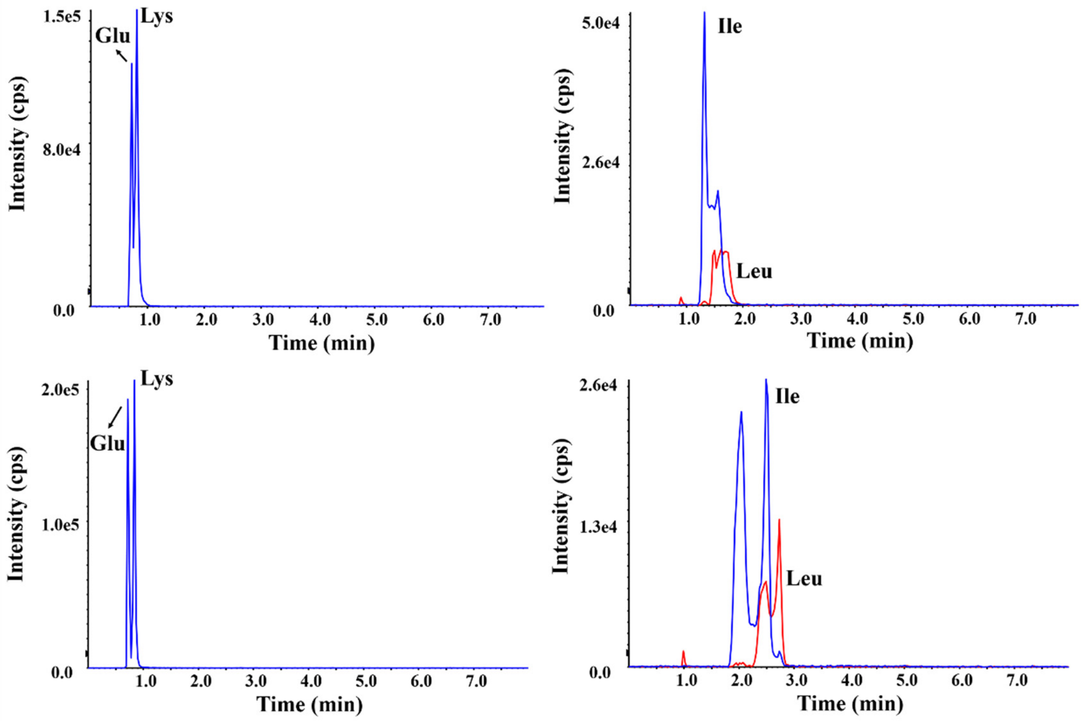

Figure S4. Retention performance of two pairs of isomers on Polar C_{18} (upper two graphs) and HSS T3 column (bottom two graphs).

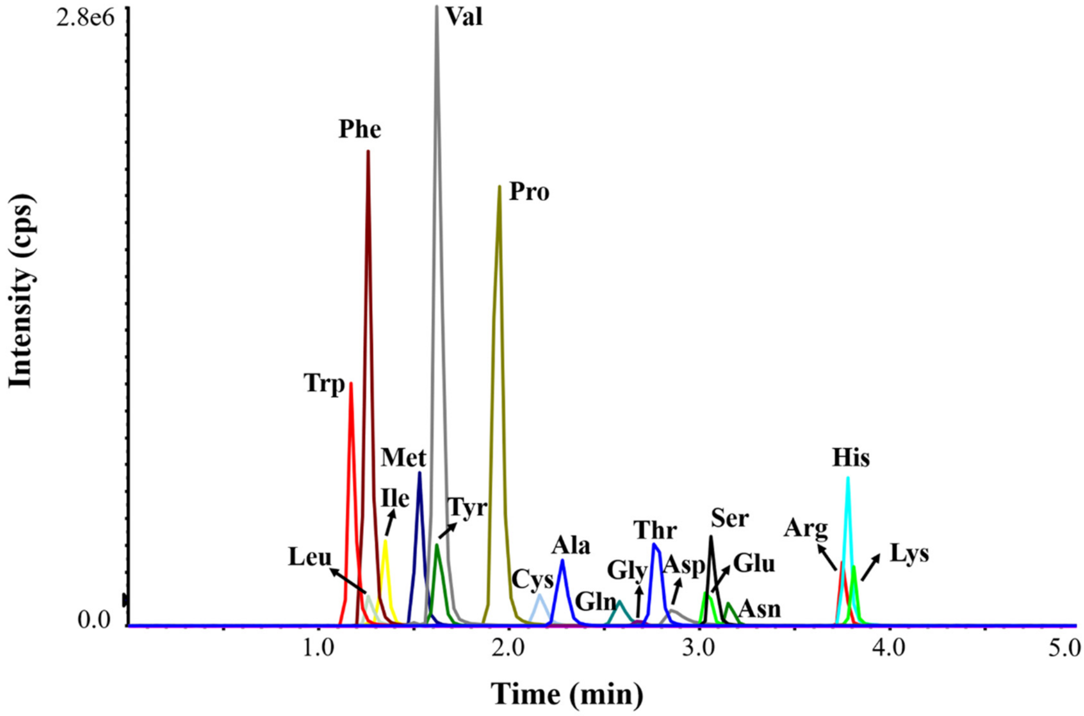

Figure S5. Chromatogram of 20 FAAs using 5 min gradient elution.

Table S1 MS/MS parameters for 20 FAAs.

Analyte	Precursor ion	Product ion	DP (V)	CE (V)
Ala	90.1	44.2	30	18
Arg	175.1	70.1	50	30
Asn	133.1	74.1	40	18
Asp	134.1	74.1	40	17
Cys	122.1	59.1	40	31
Gln	147.1	84.1	35	16
Glu	148.1	84.1	50	25
Gly	76.0	30.3	30	15
His	156.1	110.1	40	23
Ile	132.1	69.2	30	25
Leu	132.1	43.2	40	35
Lys	147.1	84.1	40	30
Met	150.1	104.0	30	14
Phe	166.1	120.1	32	20
Pro	116.1	70.1	40	20
Ser	106.1	60.2	35	15
Thr	120.1	74.2	35	16
Trp	205.1	188.0	50	17
Tyr	182.1	136.0	45	16
Val	118.1	72.2	30	15

Table S2 Initial LC gradient for three columns.

Chromatographic column	Gradient (flow rate 0.3 mL/min, mobile phase A=H_2O containing 0.1% FA, B=ACN containing 0.1% FA)
Polar C_{18}	0-2 min 2% B, 2-4 min 2%-80% B, 4-5 min 80% B, 5.1-8 min 2% B
HSS T3	0-2 min 2% B, 2-4 min 2%-80% B, 4-5 min 80% B, 5.1-8 min 2% B
BEH amide	0-0.5 min 80% B, 0.5-2.5 min 80%-50% B, 2.5-4 min 50% B, 4.1-8 min 80% B

Table S3 Final LC condition after optimization using BEH amide column.

Time (min)	Flow rate (mL/min)	Mobile phase B
Initial	0.4	80
0.5	0.4	80
1	0.4	50
3	0.4	50
3.1	0.4	80
5	0.4	80

Table S4 Detailed information of these ten honey samples.

Sample number	Botanical source	Producing region
1	linden honey	Fuzhou, Jiangxi province
2	linden honey	Fuzhou, Jiangxi province
3	locust honey	Ningbo, Zhejiang province
4	locust honey	Ningbo, Zhejiang province
5	jujube flower honey	Changping, Beijing
6	jujube flower honey	Changping, Beijing
7	loquat honey	Ningbo, Zhejiang province
8	loquat honey	Ningbo, Zhejiang province
9	loquat honey	Guilin, Guangxi province
10	loquat honey	Guilin, Guangxi province

Table S5 FAA content in ten honey samples.

Analyte	FAA content (μg/g)									
	Sample 1	Sample 2	Sample 3	Sample 4	Sample 5	Sample 6	Sample 7	Sample 8	Sample 9	Sample 10
Ala	5.7	5.2	5.6	5.9	8.6	9.5	2.7	2.7	2.3	3.1
Arg	0.6	0.8	1.6	2.1	1.8	2.1	2.8	2.4	3.2	3.8
Asn	1.7	1.4	1.3	1.0	1.1	0.8	0.5	0.6	0.5	0.7
Asp	5.0	4.8	6.6	5.2	7.6	9.1	2.7	3.1	2.5	3.8
Cys	-	-	-	-	-	-	-	-	-	-
Gln	1.2	0.8	2.0	1.6	5.6	4.2	4.0	3.6	3.2	4.5
Glu	3.1	3.3	5.4	4.4	4.4	6.1	2.5	2.8	2.4	3.5
Gly	1.3	0.8	1.7	1.9	0.4	0.5	1.1	1.2	1.5	1.4
His	0.7	0.6	0.9	0.8	0.5	0.4	0.9	0.7	0.8	0.9
Ile	7.5	5.9	3.4	3.6	3.8	3.7	3.0	2.3	4.0	3.6
Leu	5.6	4.8	3.1	3.3	3.6	4.7	4.4	3.5	2.9	2.8
Lys	1.7	1.6	4.0	4.2	0.8	1.2	6.1	5.2	4.8	5.9
Met	0.1	0.2	0.2	0.3	0.2	0.1	0.4	0.5	0.4	0.6
Phe	369.7	323.5	8.3	9.1	24.2	14.8	4.2	3.1	3.9	5.4
Pro	691.9	601.7	174.5	168.5	691.9	681.5	180.5	205.9	187.7	233.5
Ser	1.1	1.2	2.8	2.2	1.2	1.6	2.2	1.7	1.2	2.3
Thr	1.0	0.9	2.0	1.8	1.0	1.7	1.6	1.3	1.5	1.8
Trp	0.1	0.2	0.2	0.1	0.3	0.4	0.1	0.2	0.2	0.2
Tyr	76.9	57.7	7.7	9.2	12.1	12.5	9.3	8.1	7.5	6.9
Val	7.0	6.7	5.6	5.7	5.8	6.6	3.3	2.8	4.2	3.8

Table S6 p-value of Phe content in honey from different botanical origins

	linden honey vs locust honey	linden honey vs jujube flower honey	linden honey vs loquat honey	locust honey vs jujube flower honey	locust honey vs loquat honey	jujube flower honey vs loquat honey
p-value	0.0046	0.0051	0.0045	0.1492	0.0176	0.0787
